# Dr. G. V. Black’s Investigations of the Human Teeth

**Published:** 1896-01

**Authors:** 


### Editorial.

### DR. G. V. BLACK'S INVESTIGATIONS OF THE HUMAN TEETH.

In the judgment of the writer, any attempt to express an opinion for or against the work of any one where this has been based on actual investigation would be not only valueless but the height of folly. Dentistry has had entirely too much of this talk for talk's sake, and it has therefore been with some degree of satisfaction that we have observed that, with but one exception, the entire dental world has been silent in regard to Dr. Black's work. It is a reflection on the intelligence of dentists to have this charge made by a contemporary: "Dr. Black's paper was published in May last. Up to this writing there has been no response. Are we all paralyzed?" We prefer to think that intelligent dentists, having found the conclusions at variance with experience, have decided to wait before giving expression to their thoughts until some one with time, means, and practical knowledge could repeat these experiments, and confirm or reject them.

While this view is true, it still remains the duty of the faithful observer to note apparent errors of investigation here or elsewhere.

Viewed in any light, the labor of this very able worker in untrodden fields must be held to be most painstaking and thorough, and quite equal to any that has preceded it. This is Dr. Black's due, and while it is impossible to concede results entirely satisfactory, the fact remains that he has led the way for others to follow. The difficulties attending an investigation of this kind are innumerable, and, it is feared, will ever remain insurmountable. The controversary between "soft teeth" and "dense teeth" has not been settled by these investigations; indeed, it is probable that this will continue with increased intensity.

While desirous of giving Dr. Black due credit for all that he has done, as represented in the series of tables in the May number of the *Dental Cosmos*, it must be clear to any one dispassionately reviewing his labors that in the first paper is demonstrated but one thing, and that is that dentine becomes more dense by age.

Dr. Black states that his investigations are based on an effort to find "differences in the density, percentage of lime salts, strength, etc., occurring in human teeth, and to find what relation, if any, such differences may bear to dental caries and the diseases of the teeth." He says further, "I have chosen to confine the examination for density, proportion of water, lime salts, and organic matter to the dentine, as best expressing the character of the teeth as a whole."

For the collection of material he was forced to depend on the good offices of many professional friends. They were instructed "to place the teeth in an envelope just as they come from the mouth, write on the envelope the nativity, age, state of the general health, color of the hair, to class the teeth as bad, fair, or good as to occurrence and rapidity of caries, record the condition of the gums," etc.

There are two conditions that would seem to the general observer to invalidate the entire series of experiments. In the first place, dentine was taken as the tissue best calculated to represent density, or rather the character, of the entire tooth. The propriety of this selection may well be questioned in view of the fact that it is not dentine that is the resisting body against the attacks of caries, and that therefore inferences drawn from experiments upon this tissue must be largely fallacious; or, at least, they can have no force as antagonists to clinical observations.

The method adopted in collecting material seems faulty. It probably could never be otherwise; hence no reliance can be placed on conclusions. Teeth are "placed in an envelope" by persons, and marked "bad, fair, or good" as to progress of caries. These individuals may be thoroughly careful and competent to make the selection, but there is no evidence that such is the fact. It is in the experience of every intelligent observer that the "occurrence of caries" is no test as to the character of teeth. Those regarded in our own practice as the best in resisting power have melted with great rapidity by change of environment, and it is a notorious fact that the so-called dense teeth will decay by change of climate superinducing general physical disturbance. The difference between the great sensibility and poor character of the teeth of the Sclavonic people as compared with the Germans, closely contiguous, has demonstrated to many and to the writer that there is a wide difference in the structure of teeth not discoverable by the most exact instruments. The same diversity in structure is not confined to certain nationalities, but abounds, as a serious evil, in this country of mixed inheritances. To attempt therefore to classify the average superior quality of teeth of the German, English, or Irish by the amount of dental caries existing among these races would be simply to exhibit ignorance of conditions. Dr. Black says in explanation of this that "there has been no selection whatever of teeth for this work, but such teeth as came into my hands I used, all of them that *were reasonably perfect at the gingival line.*" Dr. Black assumes too much when he asserts that dentists have interpreted clinically teeth firm and clear in appearance, with neat gingivæ, clean interproximate spaces, and free from caries as "teeth that are well calcified," and teeth "coated with some form of glutinous deposit, perhaps, and rapidly progressive caries in numerous spots," as indicating "soft teeth or teeth that are poorly calcified."

It is difficult to conceive of a dentist so poorly skilled as to differentiate these important conditions upon such an absurd basis. That there is a marked difference in teeth, clinical observation has demonstrated from the earliest experiences in dentistry. That teeth become denser by age was known in the infancy of the profession, and while it is very satisfactory to have this demonstrated beyond cavil, the fact remains that nothing really new has been added to dental experience; indeed, it may be questioned whether these investigations have not tended to obscure knowledge already obtained and have made the path of the conscientious operator and teacher doubly difficult.

"There is no basis for the supposition that the teeth of children under the age of twelve *are too soft* to recieve metallic fillings." This is the dictum deduced from the prepared tables, and based on these it is doubtless correct, but what experienced operator ever founded his objection on teeth under twelve as being too soft if by that is meant lack of density. This term has, it is true, been used wrongly, and intended to convey the idea of immaturity. In that sense it is correct. The child of twelve and for many years thereafter has not teeth fitted to "receive metallic fillings," notwithstanding the tables presented or the high authority that produced them. These teeth are not fully developed, the tubes of the dentine are larger and consequently more readily affected by external irritants let these be from whatever source derived, whether thermal or medicinal. To deny this fact is to attack the very ground-work of dental histology, and we therefore do not for a moment suppose that Dr. Black means to be thus understood.

There is possibly very injurious results to follow from this strong statement that "there is no basis for the supposition that some teeth are too soft or too poorly calcified to bear filling with gold, . . . since all are found to be abundantly strong." Advice such as this can only result in great harm, as it antagonizes the recognized observations of the entire dental profession for the past century. No intelligent practitioner can accept it, no matter what the authority may be that supports it, for, as stated, it is not true. If this is to be the teaching of the future, the students of the new era are certainly to be pitied, for a world of difficulty and many mortifying experiences await them. There must be a discrimination made as to character of teeth, and this should be based not on instrumental tests, but on more exact and reliable observation through daily experience with all sorts and conditions of men, women, and children.

If there be the same quantity of inorganic material in teeth of the same age, why, may it be queried, do we find some teeth in which caries progresses with extreme slowness, resulting in the transparent zone of Tomes, others with a character of dentine not much above chalk in texture, decaying with great rapidity? There is no explanation for this in the tables presented, nor is any attempted beyond the very unsatisfactory assertion that environments differ. This explanation cannot be accepted.

If Dr. Black had made selections instead of declining to make any distinction between teeth, and had secured material from a mouth recognized by those competent to decide as being of the purely soft variety, and submitted these to the test which he gave to those selected indiscriminately, results would have been obtained which would have gone very far towards settling this question. In the opinion of the writer, white teeth are by no means the poorest teeth, and this view is confirmed by Dr. Black, although how the experiments prove it is not clear, as all teeth become whiter as the drying out process continues. The impossibility of securing teeth of the character mentioned must remain a bar to exact observation, but until this be done we must decline to accept the conclusions of Dr. Black, except in a modified sense.

